# A Bibliometric Analysis of 8271 Publications on Thyroid Nodules From 2000 to 2021

**DOI:** 10.3389/fendo.2022.845776

**Published:** 2022-04-21

**Authors:** Qianqian Zhang, Xiaoyan Xin, Li Wang

**Affiliations:** ^1^ Department of Health Management, School of Public Health, Hangzhou Normal University, Hangzhou, China; ^2^ Department of Endocrine and Metabolism, Zhuhai People’s Hospital, Zhuhai Hospital Affiliated with Jinan University, Zhuhai, China

**Keywords:** thyroid nodules, bibliometric, thyroid cancer, FNA (fine needle aspiration), management

## Abstract

**Introduction:**

Thyroid nodules (TNs) are a common clinical condition. The probability of thyroid nodules being malignant is 7-15%. However, in recent decades, a number of publications on TNs have not been well summarized and discussed. The aim of this study was to summarize and sort out medical publications on TNs over the past 2 decades using a bibliometric method.

**Materials and Methods:**

Medical publications from January 1^st,^ 2000, to November 1^st,^ 2021, were searched in the Web of Science Core Collection database using the Medical Subject Heading (MeSH) term “thyroid nodule”. Full associated data were downloaded, and detailed information was extracted using the bibliometric analysis platform VOSviewer.

**Results:**

A total of 8271 publications related to TNs from the last 2 decades were found and included in this study. An increasing trend was presented in the annual number of publications. The United States, China and Italy contributed the most publications. Carcinoma, management, ultrasound, and fine-needle aspiration were the most popular subjects in the field of TNs. The topics of the studies could be stratified into four clusters. The first cluster was using ultrasound to evaluate the nodules, including the thyroid imaging reporting and data system (TI-RADS), elastography and benign features. The second cluster was the fine-needle aspiration method, including the Bethesda system, cytology and BRAF mutations. The third cluster was the management of nodules, including radiofrequency and thermal ablation, surgery, and consensus statements. The last cluster was carcinoma, which is correlated with all three clusters described above. The preoperative diagnosis of cytologically indeterminate nodules was particularly highlighted in the top 10 most cited publications in recent years.

**Conclusion:**

How to diagnose thyroid nodules as malignant or benign, especially in cytologically indeterminate nodules, is still the most concerning topic in TN research. Although the fine-needle aspiration method and gene-expression classifiers show promising results, there is still a crucial need for translations from fundamental studies to clinical applications.

## Introduction

Thyroid nodules are a common clinical condition and are defined as discrete lesions within the thyroid gland. On ultrasound, thyroid nodules usually show a different echotexture from the surrounding thyroid parenchyma. Epidemiological data showed that the prevalence of palpable thyroid nodules was approximately 5% (5% in women and 1% in men) (1). However, the diagnosis rate of thyroid nodules by ultrasound (US) can be 19%–68% in adults, and women and elderly individuals tend to have a higher prevalence (2). Approximately 90% of nodules are benign and asymptomatic. However, approximately 7-15% of thyroid nodules are diagnosed as thyroid carcinoma, which makes methods to exclude carcinoma from thyroid nodules clinically important (3).

During the last 2 decades, with new diagnostic methods and minimally invasive surgery techniques arising, vital and significant progress has been accomplished in the area of thyroid nodules (4). This leads to a growing number of publications in these fields. These medical publications can replicate the vital issues of this field and guide future research directions (5).

A bibliometric analysis is often used to develop more accurate diagnostic methods and better treatment strategies. Professor Cooper and Professor Anton recently published a bibliometric analysis article in *Journal Thyroid* to assess the current status of clinical thyroidology literature (6). However, in the last 2 decades, a number of publications on TNs have not been well summarized and discussed.

The aim of this study was to summarize and sort out medical publications on TNs over the last 2 decades using a bibliometric analysis. Furthermore, to analyse the publications to a better extent, full associated data were downloaded, and detailed information was extracted using the bibliometric analysis platform VOSviewer. Hopefully, this study can provide some interesting insights into the developments that have occurred over the last twenty years in the diagnosis and management of TNs.

## Materials and Methods

Data in this study were obtained from the Web of Science Core Collection databases, which is a curated collection of high-quality scholarly content. All the included journals were selected cover-to-cover, and details such as the authors, abstracts, keywords and cited references were captured for each paper. Medical publications focusing on TNs were searched from January 1^st^, 2000, to January 1^st^, 2021, using the Medical Subject Heading (MeSH) term “thyroid nodule”. The search strategy was “TS= ((thyroid nodule) OR (Nodule, Thyroid) OR (Nodules, Thyroid) OR (Thyroid Nodules)), and the publication language was limited to English.

A total of 8271 publications related to TNs from the last 2 decades were found and included in this study. Original Research, short reports, societal guidelines and case studies were enrolled, and review articles were excluded. Full associated data were downloaded, and detailed information was extracted using the bibliometric analysis platform VOSviewer, including the publication year, keywords, abstract, authors, and author affiliations. Keywords appearing more than 10 times were included in the analyses. In addition, the top 100 concurrent keywords are presented in the figures. VOSviewer (version 1.6.6) is a widely used tool for sorting and visualizing bibliometric analysis that is run by the Centre for Science and Technology Studies at Leiden University. Analyses were made by Dr Zhang and Xin, and Professor Wang was the supervisor investigator responsible for resolving the discrepancy occurring during the sort.

Since the data of this study were obtained from a public database, ethical approval from an institutional review board or ethics committee was not required.

## Results

### Overview

**Figure 1:**
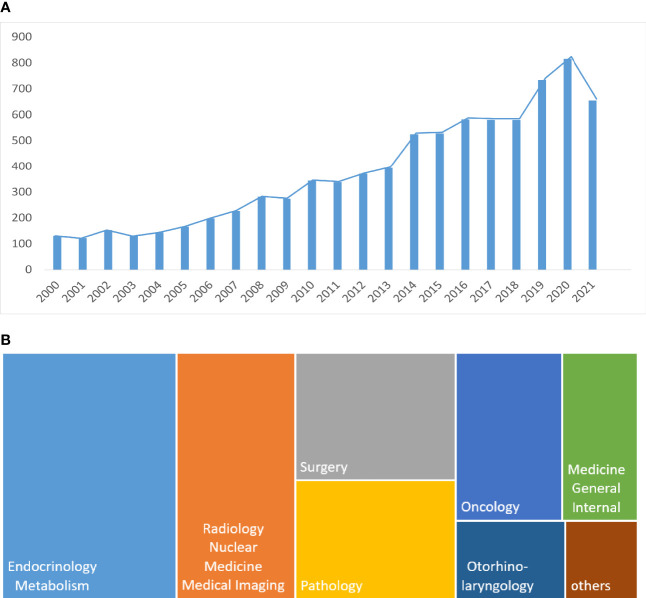
**(A)** Medical publications by year; **(B)** Research field categories involving thyroid nodules.

A total of 8271 publications related to TNs from the last 2 decades were found and included in this study. As shown in **Figure 1A**, the number of TN documents continuously increased over the past 2 decades. Since only ten months were included for 2021, the numbers dropped slightly. The COVID-19 pandemic might have partly caused this drop in numbers. The proportion of publication areas is shown in **Figure 1B**. Endocrinology, radiology, surgery, and pathology studies shared the highest proportion of publications.

### Countries

**Figure 2:**
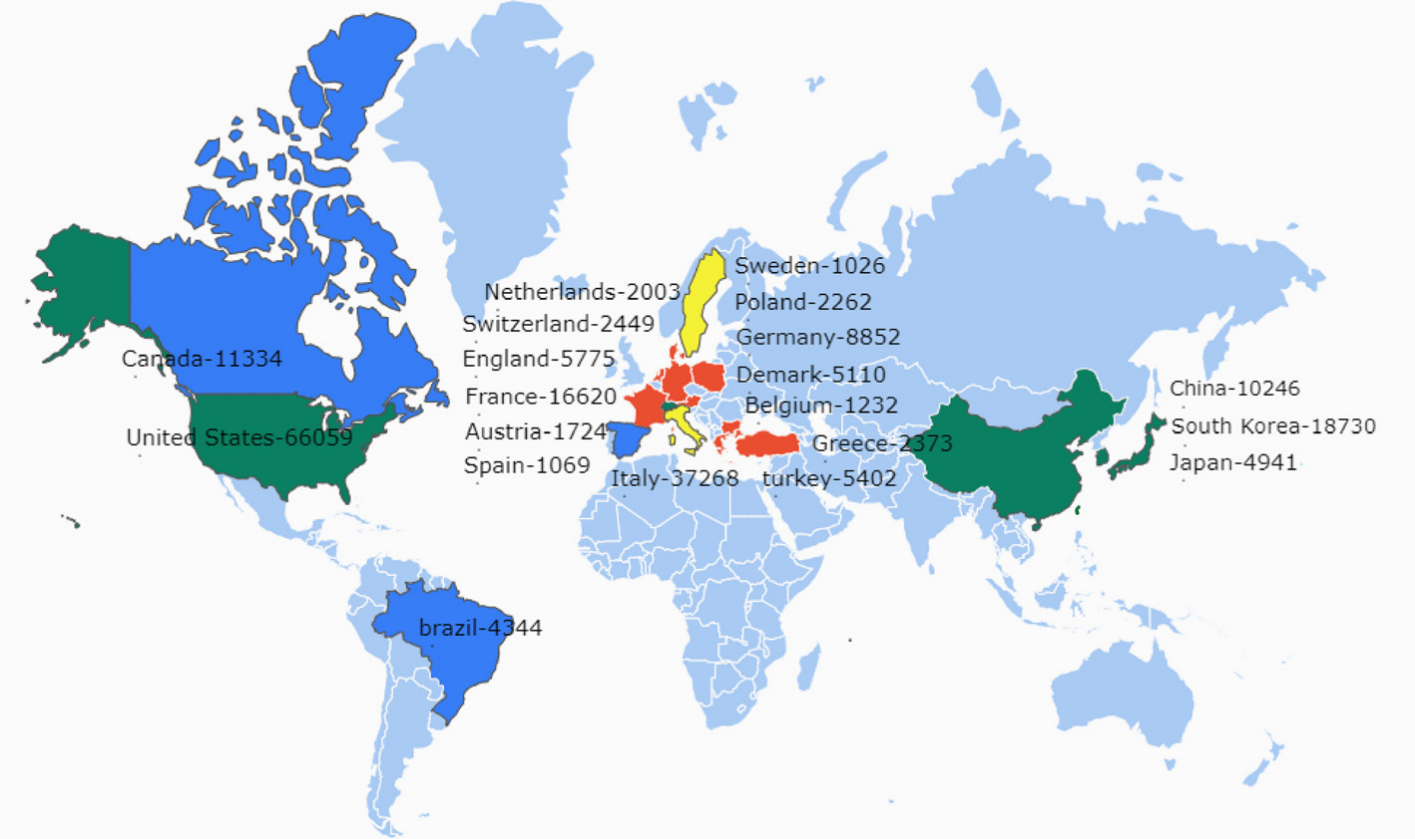
Top 20 countries with the most cited medical publications. Country name and citation number are shown on the map. The countries are sorted into different-coloured clusters and countries with the same colour show strong links by citation or co-authorships.

A country-specific publication citation map is shown in **Figure 2**. The top 20 countries are shown in the figure geographically, and the minimum number of documents from a country was 10. Countries are colour-labelled, and countries with strong interactions were classified into the same colour cluster. The total citation numbers were also labelled after the name of the country. As shown in the figure, China, Japan, South Korea, and the United States were in the same cluster and had high citation numbers of TN-related publications. European countries were also quite active in this topic (**Figure 2**). Even though the European countries were sorted into different clusters, most of them had great connections with each other.

### Keywords

**Figure 3:**
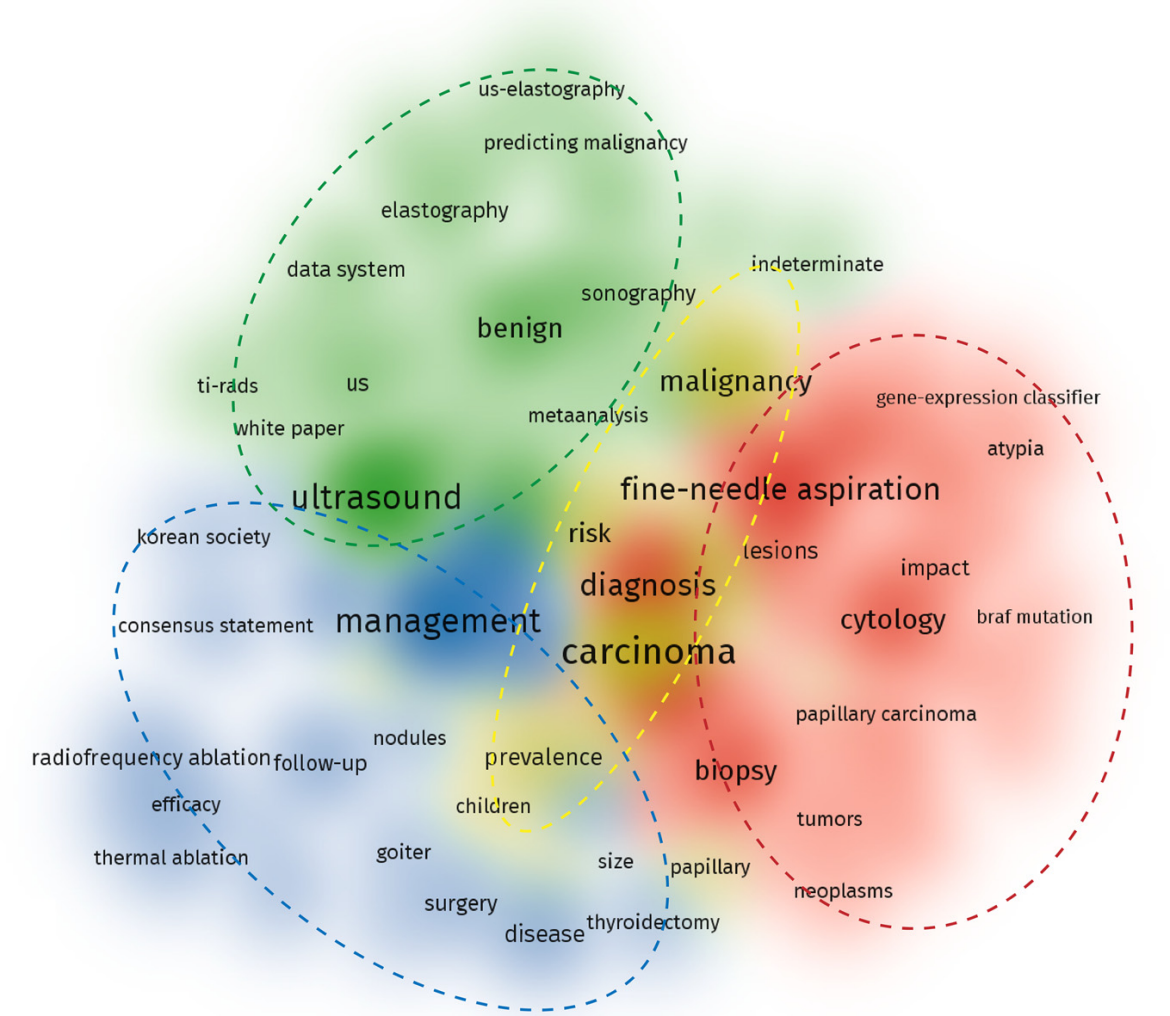
Topic clusters sorted by VOSviewer: Red, fine-needle aspiration and cytology in the diagnosis of TNs; green, ultrasound and benign features of TNs; yellow, thyroid carcinoma and malignancy of TNs; and blue, treatment and management of TNs. The gradation of circle colour represents the number of publications for each topic.

**Figure 4:**
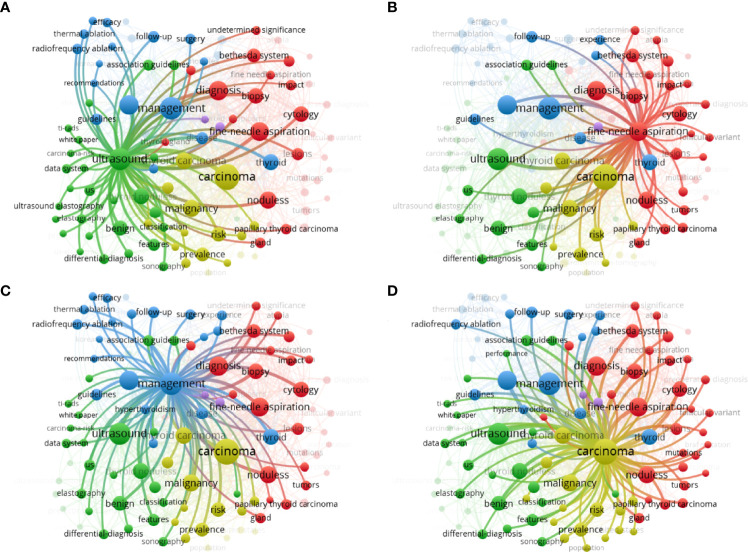
Top 100 most stated keywords represented by the visualization of citation networks. The importance of the topics is shown through the size of the circle. The weight of connections among the topics is also shown as the thickness of the line between the circles. The focalization in the blue cluster (management) **(A)**, green cluster (ultrasound) **(B)**, yellow cluster (carcinoma) **(C)** and red cluster (fine-needle aspiration and cytology) **(D)** are represented.

**Table 1** shows the top ten keywords of the study topic that received the most focus. Carcinoma, management, ultrasound, and fine-needle aspiration were the areas that received the most attention. Hence, the top 100 keyword visualization network figures were sorted into four clusters according to the theme keywords (**Figure 3**). In the figure, we can clearly see that the publications over the past 2 decades revolved around four topics. The first cluster used ultrasound to evaluate the nodules, including the thyroid imaging reporting and data system (TI-RADS), elastography and benign features. As the most important detection method of thyroid nodules, ultrasound not only has a large amount of subrelated keywords, including elastography and benign features but also involved with fine-needle aspiration as a visual guidance. According to the ATA guidelines, some thyroid carcinomas with typical malignancy features can be diagnosed directly by ultrasound. In addition, since it is suggested to perform follow-up checks every 1 or 2 years for benign nodules, ultrasound was also involved in the management of TNs (**Figure 4A**). The second cluster focused on the cytology and biopsy of nodules. Fine-needle aspiration is considered a safe and convenient way to obtain a cytological assessment of thyroid nodules, and the circle has a strong relation with diagnosis and management. As recommended by the 2015 ATA guidelines, the Bethesda classification also plays a vital role (**Figure 4B**). The third cluster involved the management of nodules, including radiofrequency and thermal ablation, surgery, and consensus statements (**Figure 4C**). The last cluster is carcinoma, since the most vital task is to diagnose malignancy and benignity of TNs (**Figure 4D**). This cluster is correlative with all three clusters mentioned above. 

**Table 1:** Top ten keywords of the study topic most focused on related to thyroid nodules.

Keywords	Occurrence	Total link strength
**Carcinoma**	2599	6273
**Management**	1876	5238
**Ultrasound**	1753	4869
**Fine-needle aspiration**	1125	3512
**Diagnosis**	1206	3391
**Thyroid nodules**	1246	3111
**Malignancy**	902	2994
**Nodules**	913	2581
**Biopsy**	718	2499
**Cytology**	687	2329

### Timeline

**Figure 5:**
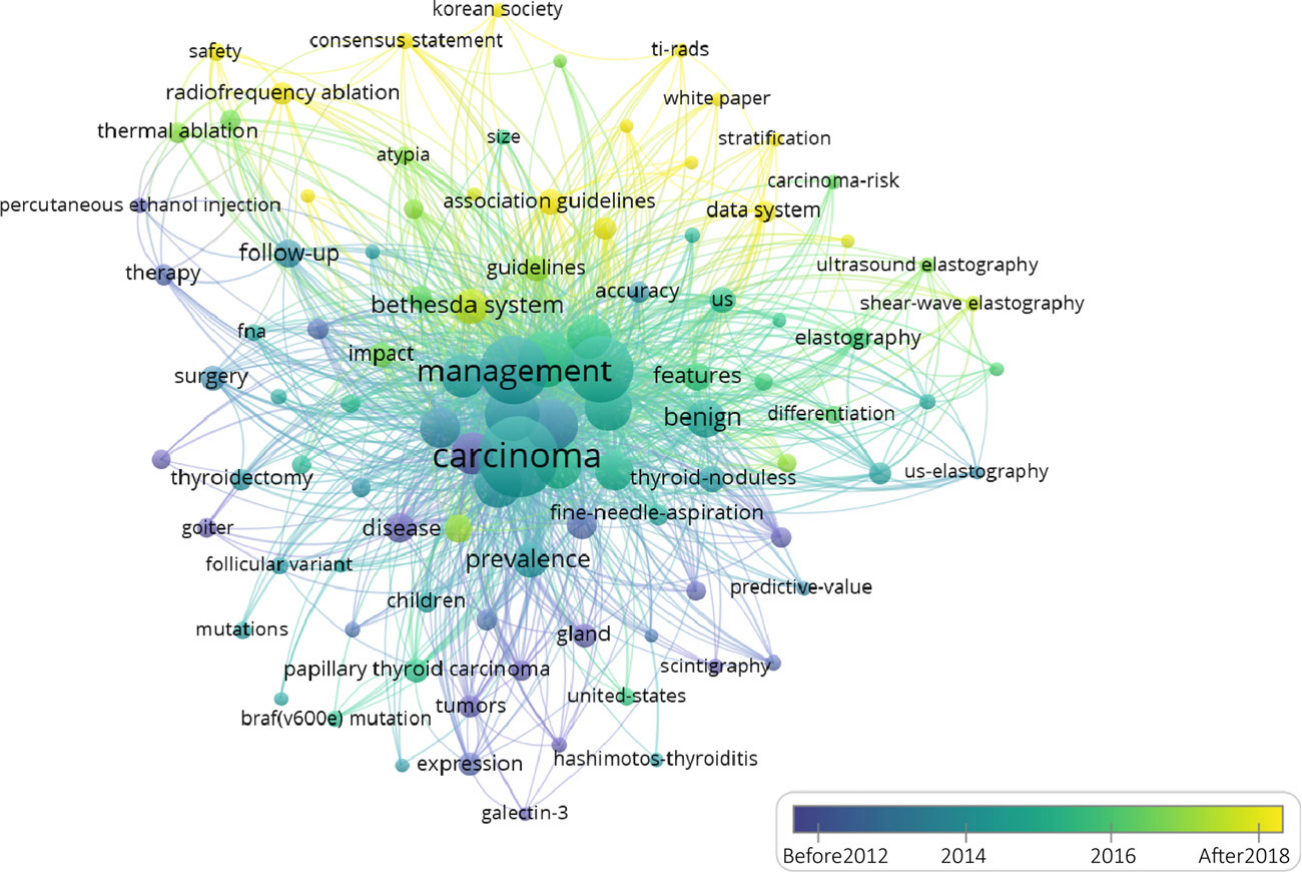
The overlay visualization by publication time of the frontier topics in the top 100 most-cited TN articles from 2000 to 2021.

The keywords were analysed by VOSviewer by timeline over the past 2 decades. As shown in **Figure 5**, the interrelations between the thyroid and other diseases, such as Hashimoto’s disease (HT) and thyroid goiters, were the focus of the first decade. Surgery and thyroidectomy were also given more attention in the early decade. With the discovery of new diagnostic and treatment approaches in recent years, TI-RAD, the Bethesda system, ultrasound elastography and radiofrequency ablation have gained increasing attention.

### Impact Assessment and Citation Analysis

The most cited article was titled “2015 American Thyroid Association Management Guidelines for Adult Patients with Thyroid Nodules and Differentiated Thyroid Cancer The American Thyroid Association Guidelines Task Force on Thyroid Nodules and Differentiated Thyroid Cancer” published in 2016 on Journal Thyroid, which is the most cited Journal (1). Professor Baek JH (Jung-Hwan) from Department radiology in Seoul National University Hospital in South Korea published 176 articles during last 2 decades mainly focused on the area of ultrasonographic (US) in the illustration of benign and malignant TN. His most cited paper was titled “Benign and malignant thyroid nodules: US differentiation - multi-center retrospective study.” This paper can be considered the early work in the diagnostic accuracy of ultrasonographic (US) in the depiction of benign and malignant TN (7).

## Discussion

This study was the first to enroll 8271 medical publications regarding TNs over the past two decades by bibliometric analysis. The number of articles published about TNs is increasing year by year and is not limited to the endocrinology and metabolism subject area. Carcinoma, management, ultrasound, and fine-needle aspiration were the most popular subjects. The most protuberant documents in a research area are those with the highest number of citations. Thus, citation analysis provides us a new angle to detect the influence of some documents on a certain topic. As we can see in the top 10 most cited publications in recent years (1, 7–15). Other than the guidelines of diagnosing and managing the benign and malignant thyroid nodules, the preoperative diagnosis of cytologically indeterminate nodules and TN in particular population (Pregnancy/postpartum women) also gained researchers attentions. Interestingly, the role for thyroid nodule screening in the general population is debatable. The main challenge in managing TNs is to identify those that are malignant. However, how to diagnose accurately without bring inappropriate excess use of US, FNA or surgery remains unclear.

In the thyroid nodule field, the most important topic is to distinguish between benign nodules and malignant nodules. As the primary method to evaluate thyroid nodules, ultrasound technology has an irreplaceable role in this area. Its application spread from the diagnosis to the management of TNs. Hence, it was highlighted in the results in this study.

With minimal harm from radiation, ultrasound is recommended by the TN guidelines for the first screening. With the increasing incidence of thyroid cancer in recent years (16), to provide specific preventive care services for patients without obvious clinical signs of thyroid carcinoma, the US Preventive Services Task Force (USPSTF) made recommendations to screen for thyroid cancer (17). Patients with TNs are all recommended to undergo ultrasonography of the thyroid to record the number, size, and characteristics of the thyroid nodules and assess cervical lymphadenopathy (8). However, the standard description of malignancy and benign nodules was not created until 2009. Professional societies and investigators have proposed ultrasound-based risk stratification systems to identify nodules, including the Thyroid Imaging, Reporting and Data System (TI-RADS). The TI-RADS was finally proposed and standardized by the ACR in 2012, which provides guidance regarding the management of thyroid nodules based on their ultrasound appearances (18).

Patients with nodules that have a malignancy risk need a biopsy to diagnose accurately. Before fine-needle aspiration (FNA) occur, partial thyroidectomy is a common method to gain the biopsy. FNA is usually performed under ultrasonographic guidance. It is the most cost-effective and harmless method to assess the nature of thyroid nodules with uncertain ultrasound descriptions (19). The ATA guidelines recommend FNA for nodules with a diameter ≥1 cm with a high or median suspicious pattern on sonography, nodules with a diameter ≥1.5 cm with a low suspicious pattern on sonography, or nodules with a diameter ≥2 cm with a very low suspicious pattern on sonography (1). An experienced cytologist was asked to evaluate the FNA samples according to the Bethesda classification system (20). Indeterminate cytology is the most challenging problem in FNA. The results of FNA can only describe the cellular morphology of the biopsy tissue. However, carcinomas diagnosed evidences include the invasion of the nodular capsule and/or vascular invasion. Considering of this limitation of FNA, a diagnostic lobectomy has to be performed eventually. Molecular analysis of FNA including the Gene Expression Classifier and gene mutations (e.g., Braf and/or TERT mutations) is likely to improve decisions for nodule surgery (21–23).

As shown in the results figures, the management of TNs was associated with the other three clusters. The reason is that TN management strategies are largely dependent on the results of ultrasonographic findings and FNA cytologic findings (24). If an eligible sample was defined as benign based on FNA and ultrasonographic findings, the recommended management was repeat ultrasonography in 1 to 2 years (25). A longer interval time (3 years) was also suggested recently (26). However, during repeat ultrasonography, if evidence of nodule growth (>50% change in volume or an increase of ≥2 mm in nodule dimensions) was detected, repeat FNA is recommended (27). If a nodule shows a specific oncogene irregularity and high positive molecular evidence for malignancy using FNA, total thyroidectomy is normally recommended. As we can see in the results, the overtreatment of TN gained lots attentions. In one of the Top10 most cited articles were reclassified encapsulated follicular variant of papillary thyroid carcinoma (EFVPTC) as non-invasive follicular thyroid neoplasm with papillary-like nuclear features (NIFTP) (14). Which result in a significant reduction in psychological and clinical consequences that brought by the diagnosis of TC including inappropriate thyroidectomy. As shown in **Figure 5**, the surgical trends for TN are changing from thyroidectomy to minimally invasive, such as radiofrequency ablation. Lifelong replacement of levothyroxine therapy and thyroid function monitoring often required in patients who undergo thyroidectomy (28, 29). The numbers of lobectomy increased significantly when inappropriate thyroid nodule screening in the general population. To reduce the burden on each family of TN patients and government health care system, the implementation of thyroid nodule screening in the general population should be taken with cautious.

This study highlighted the topics of ultrasound, the TI-RADS, fine-needle aspiration, the Bethesda system, cytology, and the management of nodules. Meanwhile, the top 10 most recently cited papers seemed to concern the topic of cytologically indeterminate nodules. Moreover, the repeat assessment time and the necessity of surgery have been argued by many studies. However, more well-designed, multiple-centre cohort studies are needed to address these arguments.

There were also some limitations of this study. Considering the high quality and comprehensive information of the publications, only the Web of Science Core Collection databases were searched for this bibliometric research.

## Conclusion

This study summarizes and sorts out medical publications on TNs over the last 2 decades using a bibliometric method. The results showed that diagnosing thyroid nodules as malignant or benign, especially cytologically indeterminate nodules, is still the most concerning topic in TN research. Although fine-needle aspiration and the gene-expression classifier show promising results, there is still a crucial need for translations from fundamental studies to clinical application.

## Data Availability Statement

The original contributions presented in the study are included in the article/**Supplementary Material**. Further inquiries can be directed to the corresponding author.

## Author Contributions

All authors listed have made a substantial, direct, and intellectual contribution to the work and approved it for publication.

## Conflict of Interest

The authors declare that the research was conducted in the absence of any commercial or financial relationships that could be construed as a potential conflict of interest.

## Publisher’s Note

All claims expressed in this article are solely those of the authors and do not necessarily represent those of their affiliated organizations, or those of the publisher, the editors and the reviewers. Any product that may be evaluated in this article, or claim that may be made by its manufacturer, is not guaranteed or endorsed by the publisher.
