# Corrigendum: A bibliometric analysis of 8271 publications on thyroid nodules from 2000 to 2021

**DOI:** 10.3389/fendo.2024.1505505

**Published:** 2024-10-28

**Authors:** Qianqian Zhang, Xiaoyan Xin, Li Wang

**Affiliations:** ^1^ Department of Health Management, School of Public Health, Hangzhou Normal University, Hangzhou, China; ^2^ Department of Endocrine and Metabolism, Zhuhai People’s Hospital, Zhuhai Hospital Affiliated with Jinan University, Zhuhai, China

**Keywords:** thyroid nodules, bibliometric, thyroid cancer, FNA (fine needle aspiration), management

In the published article, there was an error in affiliations order. Instead of “1Department of Endocrine and Metabolism, Zhuhai People’s Hospital, Zhuhai Hospital Affiliated with Jinan University, Zhuhai, China; 2Department of Health Management, School of Public Health, Hangzhou Normal University, Hangzhou, China”, it should be “1Department of Health Management, School of Public Health, Hangzhou Normal University, Hangzhou, China; 2Department of Endocrine and Metabolism, Zhuhai People’s Hospital, Zhuhai Hospital Affiliated with Jinan University, Zhuhai, China”.

The authors apologize for this error and state that this does not change the scientific conclusions of the article in any way. The original article has been updated.

