# Supplementary figures and images for: A Bibliometric Analysis of 8271 Publications on Thyroid Nodules From 2000 to 2021
Source: Front Endocrinol (Lausanne). 2022 Apr 21;13:845776. doi: 10.3389/fendo.2022.845776 (PMC9068984; doi:10.3389/fendo.2022.845776)

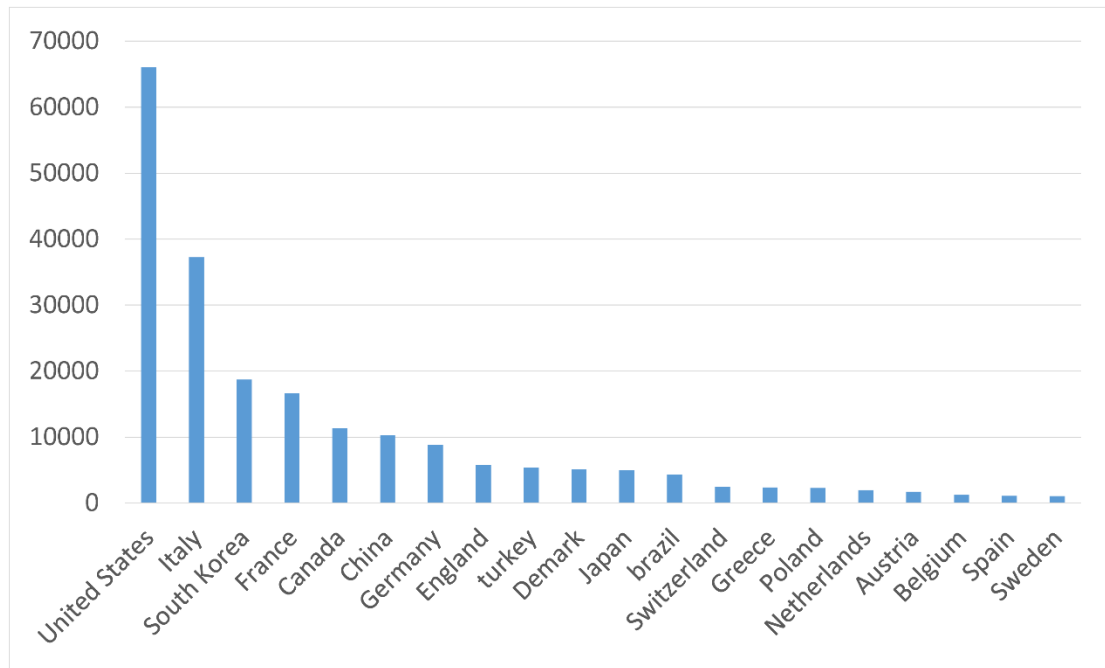

Supplementary figure1:Top 20 countries with the most cited medical publications.

Supplement: Supplementary file 1 [file Image_1.pdf]
